# Epidemiology and associated risk factors of giardiasis in a peri-urban setting in New South Wales Australia

**DOI:** 10.1017/S0950268818002637

**Published:** 2018-09-28

**Authors:** P. Zajaczkowski, S. Mazumdar, S. Conaty, J. T. Ellis, S. M. Fletcher-Lartey

**Affiliations:** 1 Faculty of Science, School of Life Sciences, University of Technology Sydney, Australia; 2 Healthy People and Places Unit, South Western Sydney Local Health District, Liverpool, Australia; 3 Public Health Unit, South Western Sydney Local Health District, Liverpool, Australia

**Keywords:** Control, diarrhoea, epidemiology, giardiasis, surveillance, transmission

## Abstract

Giardiasis is one of the most important non-viral causes of human diarrhoea. Yet, little is known about the epidemiology of giardiasis in the context of developed countries such as Australia and there is a limited information about local sources of exposure to inform prevention strategies in New South Wales. This study aimed to (1) describe the epidemiology of giardiasis and (2) identify potential modifiable risk factors associated with giardiasis that are unique to south-western Sydney, Australia. A 1:2 matched case-control study of 190 confirmed giardiasis cases notified to the South-Western Local Health District Public Health Unit from January to December 2016 was employed to investigate the risk factors for giardiasis. Two groups of controls were selected to increase response rate; Pertussis cases and neighbourhood (NBH) controls. A matched analysis was carried out for both control groups separately. Variables with a significant odds ratio (OR) in the univariate analysis were placed into a multivariable regression for each matched group, respectively. In the regression model with the NBH controls, age and sex were controlled as potential confounders. Identified risk factors included being under 5 years of age (aOR = 7.08; 95% confidence intervals (CI) 1.02–49.36), having a household member diagnosed with a gastrointestinal illness (aOR = 15.89; 95% CI 1.53–164.60) and having contact with farm animals, domestic animals or wildlife (aOR = 3.03; 95% CI 1.08–8.54). Cases that travelled overseas were at increased risk of infection (aOR = 19.89; 95% CI 2.00–197.37) when compared with Pertussis cases. This study provides an update on the epidemiology and associated risk factors of a neglected tropical disease, which can inform enhanced surveillance and prevention strategies in the developed metropolitan areas.

## Introduction

*Giardia duodenalis* (also known as *Giardia lamblia* or *Giardia intestinalis*) is one of the most common enteroparasites affecting humans with an estimated 280 million people being infected each year, around the world [1]. It is a protozoan parasite that causes infection in the bowel and clinically manifests as a diarrhoeal illness. Additionally, giardiasis has been associated with the development of chronic diarrhoea or irritable bowel syndrome, debilitating fatigue and reactive arthritis [2]. Giardiasis is not a life-threatening disease, however, infections may often go unnoticed due to many cases having a lack of symptoms. If left without treatment, the infection can become serious; impairing the development of children and resulting in a failure to thrive [3]. Certainly, giardiasis contributes negatively to public health development of endemic countries and causes devastating socio-economic loss. In 2004, *G. duodenalis* was officially included in the WHO Neglected Diseases Initiative [4]. Meanwhile, in Australia, giardiasis is a notifiable disease in several states and territories including New South Wales (NSW) [5].

Giardiasis is the most common notifiable parasitic infection in NSW. While the burden of disease is greater in developing settings with poor access to water, sanitation and hygiene (WASH) facilities, sporadic cases occur in developed countries including Australia and outbreaks are not uncommon [6]. In 2014, nearly 3000 cases were notified by laboratories in NSW [7] and 3434 cases reported in 2015 [7]. South Western Sydney (SWS) accounts for approximately 6% of cases state-wide. Amongst hospitalised patients, giardiasis was the second most commonly identified enteric protozoa, affecting mainly school age and young children [8]. In Australia, giardiasis is frequently associated with waterborne infections, day care centre disease outbreaks and travel-associated diarrhoea.

Few Australian studies have documented the prevalence of giardiasis; however, there are no recent studies that have examined the risk factors that drive local transmission of giardiasis [9, 10]. The aim of this study was to describe the epidemiology of giardiasis and to identify the risk factors and sources of exposure associated with the disease in the SWS region of NSW. The study provides information on the impact of giardiasis on human health in SWS and a better understanding of the epidemiology and associated risk factors that can inform public health control strategies.

## Materials and methods

### Study site

The South-Western Sydney Local Health District (SWSLHD) was the research site. The SWSLHD includes seven Local Government Areas (LGA): Bankstown, Camden, Campbelltown, Fairfield, Liverpool, Wingecarribee and Wollondilly (see Supplementary Fig. S1).

The SWSLHD is the largest and fastest growing District in metropolitan Sydney. It has a large population of approximately 900 000, has a diverse geography, including significant populations in both rural and urban areas and approximately 46% of the population speak a language other than English at home. Public Health surveillance data can provide an example of what could be occurring across the NSW state.

### Study design and data collection

#### Case-control survey

A 1:2 case-control study of risk factors was designed with the prospective recruitment of cases and controls. Cases were all confirmed cases of giardiasis notified to the SWSLHD Public Health Unit (PHU) from 1 January 2016 to 31 December 2016. A study questionnaire was developed based on a comprehensive review of the literature and was used to collect data from all cases and controls who agreed to participate in the study. Both case and control questionnaires are accessible online as Supplementary Material on the Cambridge Core website. The questionnaire asked about various socio-demographic features, self-reported clinical symptoms, information about care seeking behaviour and treatment received, the number of household members or other close contacts with similar symptoms and a range of exposures experienced 3 weeks before illness onset (for cases) or a similar time frame for controls. Enhanced data collection for this study also included additional details on potential confounders including country of birth, language spoken at home, highest educational attainment and occupation of the parents (for cases residing with their parents).

#### Recruitment and selection of participants

Laboratories are required under the *NSW Public Health Act 2010* to notify PHUs of cases of giardiasis. As per the NSW Control Guideline protocols for investigation, once a giardiasis case was notified to the SWSLHD PHU, staff contacted the diagnosing doctor of the giardiasis case to request permission to contact the case or the parent or guardian (for persons under 16-years-old), to interview the case.

#### Cases

A ‘case’ was a person who had laboratory definitive evidence for the detection of *G. duodenalis* cysts or trophozoites in stool samples or samples of duodenal contents. Informed consent was provided by the case or their parent (for persons under 16 years); with parents/guardians asked to complete the responses on behalf of children 12-years-old or younger and to provide consent for children 13–15 years to answer their own questions.

#### Controls

A ‘control’ was defined as a person resident in SWSLHD and who did not have a history of a positive *Giardia* test in the previous 3 months (due to the possibility of chronic infection with *Giardia*). In order to improve the response rate and reduce selection bias, three different sets of controls were identified for the study.

(1) Control group 1: Neighbourhood controls (NBH):
  Confirmed giardiasis cases were grouped into (i) urban and (ii) regional areas based on Australian Bureau of Standards regional classification. The aim was to identify 10 controls for each case to increase the likelihood of at least one household responding to the questionnaire. The following sampling strategy was employed.
  (i) Urban: A list of all addresses in SWSLHD geocoded to latitude-longitude coordinates was obtained from the Geocoded National Address File. This dataset is available for free from ‘Public Sector Mapping Agencies’ Australia. A 500 m radius buffer (due to the dense population in urban areas) was drawn around each case's address using Geographic Information System tools (e.g. see Supplementary Fig. S2). Ten houses were then randomly selected from the list of addresses for each buffer.
  (ii) Rural: The procedure followed was the same as for urban areas, except that 5 km buffers were used to account for population sparseness.
    A letter with the Patient Information Statement and control questionnaire were sent to the selected household, with a request that the person with the next birthday in the household complete the questionnaire. The completed questionnaire was to be returned by post in the self-addressed envelope provided.
(2) Control group 2: Pertussis case:
  Confirmed Pertussis cases notified in the same year, within the same age range (±5 years), residing within the same LGA but not on the same street as the corresponding giardiasis case were identified. If there were two or more persons meeting the criteria, one would be selected by simple random sampling using a random sampling function in Excel. Where no age match was available for the same LGA, one was selected from the closest LGA. Each control was contacted by telephone and once consent was obtained, the individual was interviewed with the standardised control questionnaire. If the person refused to participate in the study or was uncontactable after three phone calls, then the person was listed as a non-response.
(3) Control group 3: Friend Control:
  This recruitment method yielded no controls and was not considered further.

#### Sample size

Based on surveillance data, it was estimated that the SWSLHD PHU received an average of 147 giardiasis notifications annually between the years 2012 and 2015. In a 1:3 unmatched design with a two-sided confidence level of 95% (*z*_*α*/2_ = 1.96) with power (*z*_*β*_ = 0.80) of 80% and an estimated prevalence of a risk factor of 17% in controls and 40% in cases, at least 35 cases and 105 controls were needed to detect a significant risk of exposure (odds ratio (OR) >3.25) [11]. Oversampling of cases and controls was performed to accommodate for any non-responses or incompleteness in the data. As such, a total of 50 cases and 150 controls were needed.

#### Matched case-control analysis

Survey data were entered into an outbreak questionnaire developed using the Notifiable Conditions Information Management System (NCIMS) and analysed using IBM SPSS Statistics version 23.0 [12]. Pertussis cases were matched to cases by age (±5 years) and location; NBH controls were matched to cases by location (urban or rural). Univariate analysis was carried out to compare cases with each control group separately and an adjusted estimate of the OR and their 95% confidence intervals (CI) were calculated from matched pairs of cases and controls for various risk factors.

For each case-control group, variables with a significant OR in the univariate analysis were placed into a multivariable regression for each matched group respectively. No potential confounders were identified in the regression model with the Pertussis cases. In the regression model with the NBH controls, age and sex were controlled as potential confounders. A backward stepwise elimination process was employed, using a likelihood ratio test to produce the most parsimonious model [13]. All variables with a Wald *χ*^2^ statistically significant at the *P*-value of <0.05 were considered significant. OR and 95% CI for the association were reported. Cases for whom we could not identify suitable matching control subjects were excluded from the matched case-control analysis.

## Results

**Fig. 1.**
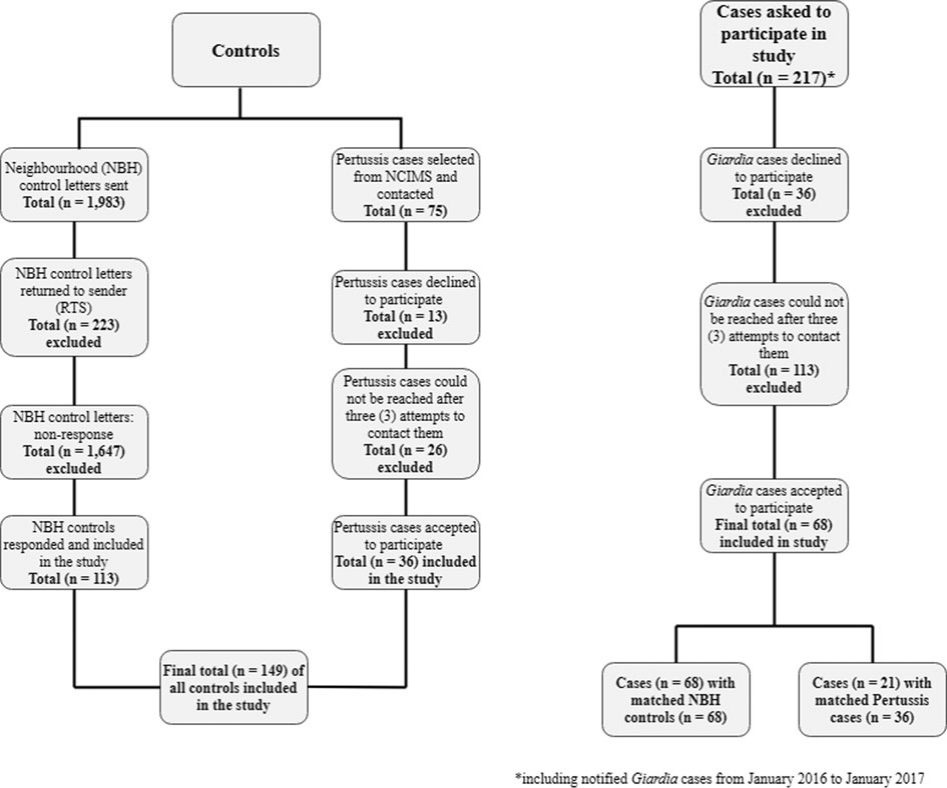
A flowchart summary of the two different control types (neighbourhood control and Pertussis case) and the number of cases used in the study.

Of the 217 giardiasis cases invited to participate in the study, 68 (31.3%) consented to be interviewed for the study (see Fig. 1). Letters were mailed to 1983 randomly selected households residing in the same neighbourhood as cases (Fig. 1). Of these, 113 controls (5.7%) returned a completed questionnaire and were included in the study. A total of 75 Pertussis cases were selected from NCIMS and contacted via telephone. Of these, 36 (48.0%) agreed to be interviewed for the study and, 26 (34.7%) could not be contacted after three telephone call attempts. To reduce the risk of selection bias, two separate matched analyses were done: one which combined 21 cases and 36 Pertussis cases and the other matched 68 cases and 68 NBH controls.

### Demographic characteristics

The distribution of the cases and controls by age and gender is presented in Table 1. Cases and controls were similar with regard to language spoken at home, highest level of education and indigenous status. Cases and controls mainly originated from urban areas in SWS as opposed to rural. More than half of case-patients (40 or 58.8%), compared with 27 (40.3%) NBH controls and 15 (41.7%) Pertussis cases were males. The age distributions varied between cases and controls with the median age being 8 (±19.4) years for giardiasis cases, 58 (±20.8) years for NBH controls and for Pertussis cases, 8 (±17.9) years (see Table 1).

**Table 1.** Demographics of cases and controls

Demographics	Cases % (*N* = 68)	Neighbourhood controls % (*N* = 113)	*P*-value	Cases % (*N* = 21)	Pertussis cases (*N* = 36)	*P*-value
Median age in years (range)	8.0 (Range 0–83; Mean 18.3 (13.6–23.0) ± 19.36)	57.5 (Range 0–84; Mean 51.0 (46.0–56.1) ± 20.8)	NR^a^	7.0 (Range 1–70; Mean 19.4 (10.0–28.9) ± 20.8)	8.0 (Range 1–65; Mean 16.3 (10.2–22.3) ± 17.9)	NR
0–4 years	36.8% (25)	2.9% (2)	0.001*	28.6% (6)	41.7% (15)	0.242
5 years or older	63.2% (43)	97.1% (66)		71.4% (15)	58.3% (21)	
Gender						
Male	58.8% (40)	39.7% (27)	0.027*	47.6% (10)	41.7% (15)	0.662
Female	41.2% (28)	60.3% (41)		52.4% (11)	58.3% (21)	
Residence						
Urban	86.8% (59)	86.8% (59)	1.000	76.2% (16)	75.0% (27)	0.920
Rural	13.2% (9)	13.2% (9)		23.8% (5)	25.0% (9)	
Language spoken at home						
English	86.6% (58)	100.0% (10)	NR	76.2% (16)	100.0% (4)	NR
Arabic	3.0% (2)	0.0% (0)		0.0% (0)	0.0% (0)	
Hindi	3.0% (2)	0.0% (0)		9.5% (2)	0.0% (0)	
Other languages^b^	7.4% (5)	0.0% (0)		14.3% (3)	0.0% (0)	
Aboriginality						
Aboriginal but not Torres Strait Islander	0.0% (0)	1.5% (1)	NR	0.0% (0)	2.8% (1)	NR
Not Aboriginal and Torres Strait Islander	98.5% (67)	83.8% (57)		95.2% (20)	97.2% (35)	
Both Aboriginal and Torres Strait Islander	1.5% (1)	0.0% (0)		4.8% (1)	0.0% (0)	
Highest Level of Education^c^						
No formal education	4.4% (3)	2.9% (2)	NR	4.8% (1)	0.0% (0)	NR
Primary or elementary school (Year K-6 or equivalent)	1.5% (1)	4.4% (3)		4.8% (1)	2.8% (1)	
Secondary school (Year 7–12 or equivalent)	22.1% (15)	22.1% (15)		9.5% (2)	41.7% (15)	
Vocational (e.g. TAFE or skills training)	27.9% (19)	33.8% (23)		28.6% (6)	25.0% (9)	
University	36.8% (25)	35.3% (24)		42.9% (9)	30.6% (11)	
Other form of education	7.3% (5)	1.5% (1)		9.5% (2)	0.0% (0)	

a NR not reported and/or calculated.

b Other languages spoken by one person each: Bengali, Cantonese, Macedonian, Mandarin and Spanish.

c If the case was under 12 years of age, the educational level was provided by the parent/head of household answering the survey.

* Statistically significant (*P* < 0.05).

In comparison with the cases, there were significantly fewer NBH controls aged 0–4 years (36.8% *vs* 2.9%). Conversely, significantly more Pertussis cases were aged 0–4 years (28.6% *vs* 41.7%). There were also significantly more older females as NBH controls in comparison with the Pertussis cases which had significantly more children aged <5 years.

Univariate analyses revealed that males were significantly more likely to be cases when compared with NBH controls, hence sex was controlled as a potential confounder in the multivariable analysis. When controlling for sex in the multivariable analysis, cases aged under 5 years had a seven times greater risk of *Giardia* infection (aOR = 7.08; 95% CI 1.02–49.36) when compared with NBH controls. There was no difference between the ages and genders of giardiasis cases and Pertussis cases.

### Risk factors for giardiasis

Univariate analysis of the comparison between NBH controls and cases revealed that cases who, (a) were males aged under 5 years, (b) visited their/parent's country of birth, (c) had a child that attends childcare, (d) had a household member diagnosed with a gastrointestinal illness, (e) were individuals who swim in pools, (f) had contact with domestic animals, wildlife or livestock and (g) were individuals who visited a farm, zoo or wildlife park, were at increased risk for giardiasis (*P* < 0.05) (Table 2). Those who temporarily stored their water in jars, bottles or cisterns at home and for those who consumed green salad or lettuce on a daily basis were at a decreased risk (*P* < 0.05) (Table 2). When age and location were controlled in the multivariable analysis, all variables lost their significance except for having a member of household diagnosed with a gastrointestinal illness and having contact with the farm, domestic or wild animals. Those who reported swimming in pools had an elevated risk, but this was not significant (*P* = 0.06) (Table 2).

**Table 2.** Univariate and multivariable analysis of risk factors for *G. duodenalis* infection

Risk Factors	Cases % (*N* = 68)	Neighbourhood Controls % (*N* = 68)	Unadjusted OR^a^ (95% CI)	Adjusted OR^b^ (95% CI)	Cases % (*N* = 21)	Pertussis cases % (*N* = 36)	Unadjusted OR (95% CI)	Adjusted OR^c^ (95% CI)
Gender								
Male	58.8% (40)	40.3% (27)	2.17 (1.09–4.30)*	1.31 (0.47–3.67)	47.6% (10)	41.7% (15)	1.27 (0.43–3.76)	NR^d^
Female	41.2% (28)	60.3% (41)			52.4% (11)	58.3% (21)		
Age Category								
0–4 years	36.8% (25)	2.9% (2)	19.19 (4.32–85.18)*	7.08 (1.02–49.36)*	28.6% (6)	41.7% (15)	0.56 (0.18–1.78)	NR
5 years or older	63.2% (43)	97.1% (66)			71.4% (15)	58.3% (21)		
Travel within Australia								
Yes	8.8% (6)	10.8% (7)	0.80 (0.25–2.53)	NR	9.5% (2)	8.3% (3)	1.16 (0.18–7.56)	NR
Travel overseas								
Yes	19.1% (13)	11.9% (8)	1.74 (0.67–4.53)	NR	23.8% (5)	2.8% (1)	10.94 (1.18–101.41)*	19.89 (2.00–197.37)*
Visit country of birth or parent's country of birth								
Yes	76.9% (10)	18.8% (3)	14.44 (2.39–87.40)*	NR	80.0% (4)	0.0% (0)	NR	NR
Countries visited overseas								
South & Southeast Asia	38.5% (5)	37.5% (3)	NR	NR	20.0% (1)	100.0% (1)	NR	NR
West Central Asia/North Africa	7.7% (1)	12.5% (1)			20.0% (1)	0.0% (0)		
Oceania	30.8% (4)	25.0% (2)			40.0% (2)	0.0% (0)		
Europe	0.0% (0)	12.5% (1)			0.0% (0)	0.0% (0)		
Latin America & Caribbean	7.7% (1)	12.5% (1)			0.0% (0)	0.0% (0)		
Multiple Regions	15.4% (2)	0.0% (0)			20.0% (1)	0.0% (0)		
Camp or caravan								
Yes	9.0% (6)	10.4% (7)	0.84 (0.27–2.66)	NR	9.5% (2)	0.0% (0)	NR	NR
Children at home attending childcare								
Yes	50.0% (34)	7.7% (5)	12.00 (4.29–33.57)*	2.46 (0.63–9.70)	42.9% (9)	30.6% (11)	1.71 (0.56–5.21)	NR
Member of household diagnosed with a gastrointestinal illness								
Yes	24.2% (16)	1.5% (1)	21.44 (2.75–167.08)*	15.89 (1.53–164.60)*	21.1% (4)	5.6% (2)	4.53 (0.75–27.50)*	NR
Unfiltered or non-boiled tap water								
Yes	65.2% (43)	73.5% (50)	NR	NR	65.0% (13)	58.3% (21)	1.33 (0.43–4.12)	NR
Filtered tap water								
Yes	45.5% (30)	33.8% (22)	NR	NR	40.0% (8)	38.9% (14)	1.05 (0.34–3.20)	NR
Sydney water connected to home								
Yes	91.7% (55)	86.8% (59)	1.68 (0.53–5.32)	NR	82.4% (14)	91.2% (31)	0.45 (0.08–2.52)	NR
Roof-harvested rain water to home								
Yes	5.9% (4)	13.2% (9)	0.41 (0.12–1.40)	NR	9.5% (2)	2.8% (1)	3.68 (0.31–43.32)	NR
Bore water or shallow well water used in home								
Yes	0.0% (0)	1.5% (1)	NR	NR	0.0% (0)	0.0% (0)	NR	NR
Tank water used in home								
Yes	22.1% (15)	10.3% (7)	2.47 (0.94–6.50)	NR	19.0% (4)	27.8% (10)	0.61 (0.17–2.27)	NR
Temporary storage of water in jars, bottles, cisterns at home								
Yes	1.6% (1)	32.4% (22)	0.03 (0.00–0.26)*	NR	0.0% (0)	2.8% (1)	NR	NR
Swimming in pool								
Yes	57.6% (38)	28.4% (19)	3.43 (1.67–7.05)*	2.63 (0.95–7.27)	52.4% (11)	52.8% (19)	0.98 (0.34–2.89)	NR
Swimming in river, lake, lagoon, pond or similar setting								
Yes	13.2% (9)	4.4% (3)	3.31 (0.85–12.79)	NR	9.5% (2)	13.9% (5)	0.65 (0.12–3.71)	NR
Swimming in the ocean								
Yes	10.3% (7)	16.2% (11)	0.60 (0.22–1.64)	NR	14.3% (3)	16.7% (6)	0.83 (0.19–3.75)	NR
Always wash hands before eating								
Yes	60.3% (41)	67.2% (45)	0.74 (0.37–1.50)	NR	61.9% (13)	69.4% (25)	0.72 (0.23–2.22)	NR
Always wash hands after playing with animals								
Yes	80.3% (49)	76.5% (52)	1.26 (0.54–2.92)	NR	76.5% (13)	74.3% (26)	1.13 (0.29–4.35)	NR
Changing nappies of child/children								
Yes	13.8% (9)	20.9% (14)	0.61 (0.24–1.52)	NR	14.3% (3)	8.3% (3)	1.83 (0.34–10.04)	NR
Engaging in sexual activity with contact with faeces								
Yes	1.8% (1)	1.5% (1)	1.20 (0.07–19.57)	NR	0.0% (0)	0.0% (0)	NR	NR
Onsite septic system at home								
Yes	12.1% (8)	14.9% (10)	0.79 (0.29–2.14)	NR	15.8% (3)	22.9% (8)	0.63 (0.15–2.74)	NR
Contact with farm/domestic animal/wildlife								
Yes	61.8% (42)	32.4% (22)	3.38 (1.67–6.84)*	3.03 (1.08–8.54)*	71.4% (15)	72.2% (26)	0.96 (0.29–3.18)	NR
Visited a farm, zoo, wildlife park								
Yes	28.4% (19)	9.1% (6)	3.96 (1.47–10.69)*	NR	19.0% (4)	38.9% (14)	0.37 (0.10–1.33)	NR
Wildlife in close proximity to house								
Yes	26.5% (18)	17.9% (12)	1.65 (0.72–3.76)	NR	14.3% (3)	41.7% (15)	0.23 (0.06–0.94)*	0.54 (0.11–2.65)
Consumes green salad/lettuce daily								
Yes	17.9% (12)	38.8% (26)	0.34 (0.16–0.76)*	0.48 (0.15–1.52)	14.3% (3)	22.2% (8)	0.58 (0.14–2.49)	NR

*Statistically significant (P < 0.05).

a Unadjusted odds ratio.

b Odds ratio from multivariable model adjusted for sex and age and all exposures that have been previously reported to be associated with giardiasis and showed a significant association (*P* < 0.05) in the univariate model.

c Odds ratio from multivariable model adjusted for sex and all exposures that have been previously reported to be associated with giardiasis and showed a significant association (*P* < 0.05) in the univariate model.

d NR not reported and/or calculated.

The univariate analysis matching cases with the second group of controls (i.e. Pertussis cases) found that giardiasis cases were more likely to have travelled overseas and had a household member diagnosed with a gastrointestinal illness. Notably, there was a negative association found between giardiasis cases and living in close proximity to wildlife. All three variables except travelling overseas and outside Australia lost their significance in the multivariable analysis (Table 2).

## Discussion

This matched case-control study represents the value of continuing to monitor giardiasis in south-western Sydney and other parts of NSW and recommends further studies to examine the genotypes in circulation and their potential for zoonotic transmission. The results from this study indicate that some common risk factors of *Giardia* infection seen in other developed countries were not found to be significant risk factors in south-western Sydney.

Notably, the multivariable analyses among cases and NBH controls and cases and Pertussis cases found no significant association between giardiasis and those using water sourced from alternative supplies such as roof-harvested rainwater (RHRW), tank water or bore wells. An overall low number of individuals reporting drinking non-municipal water long-term may lead to this lack of association [14]. However, the result is in keeping with other Australian studies that could not identify untreated RHRW tanks as sources of infection for giardiasis, which is likely due to the fact that RHRW tanks are likely to be mainly used for potable replacement for flushing toilets, washing clothes, or watering gardens [14, 15].

Furthermore, while initial univariate analyses between cases and NBH controls found a significant association between giardiasis and those who reported swimming in pools (chlorinated, salt-water or non-chlorinated) 3 months prior to illness onset, this significance was lost in the multivariable model that controlled for age and sex. This suggests there may be a relationship between age, sex and swimming that is confounding their association with giardiasis infection in this setting. On the other hand, there are multiple studies that have established the association between swimming in pools and giardiasis infection [16–18].

Giardiasis cases were also more likely to have a household member diagnosed with a gastrointestinal illness when compared with NBH controls. A similar risk found in the univariate analysis with Pertussis cases, may be due to a low response rate. Notwithstanding, studies in Turkey and other countries have reported an increased risk of infection amongst household members infected with giardiasis [19, 20]. This indicates a potential for person-to-person transmission of infection occurring within households in SWS with infected family members (or household members) serving as sources of infection. There is also the prospect of transmission through food or water prepared by the infected individual. This study emphasises the importance of screening all household members for giardiasis once a case has been diagnosed.

In this study, the multivariable analysis found a seven times greater risk of infection for those aged under 5 years. However, when compared with Pertussis cases, the risk was insignificant. While other case-control studies have observed no significant risk associated with age, it is more likely that this result is due to the small participant numbers in the Pertussis cases group. Individuals of all age groups can be infected by *G. duodenalis* although the majority of literature maintains that giardiasis is most prevalent in school-age and younger children [21, 22]. Children tend to have a higher exposure to contaminated faeces particularly in close-contact facilities such as childcare centres putting them at greater risk of infection [16, 23, 24].

While univariate analyses among cases and NBH controls observed that males were at an increased risk of giardiasis, this association lost its significance in the multivariable analysis after being controlled for sex and age and was likely due to the fact that there were overwhelmingly more females among NBH controls [25, 26].

Cases coming in contact with domestic animals, farm animals and even wildlife were at increased risk of infection when compared with NBH controls, but not when compared with Pertussis cases. The lack of significance, when compared with the Pertussis cases, may be due to a lack of difference in exposure between the two groups, hence diluting the risk. The possible role of animals as a source of *G. duodenalis* infection to humans is still unclear, although most studies agree that animals can play an indirect role in transmission [6, 27]. Molecular investigations on *G. duodenalis* and the potential for zoonotic transmission observed that humans can only be infected with human-specific assemblages (A or B) and not from animal-adapted genotypes (C–H) [28]. A possible explanation for the present results is that animals are carriers of assemblages A or B and act as vehicles for mechanical transmission to humans who come in contact with animal's faeces at parks or wildlife settings where hand-washing facilities may not be available [29], or other environmental exposures to cysts attached to the fur of domestic animals [30].

Interestingly, the vast majority (80.9%) of *G. duodenalis* cases did not report travelling overseas within the 3 months prior to illness onset suggesting that most of the giardiasis cases were locally acquired. This is the first case-control study to examine travel history amongst giardiasis cases in this setting and is consistent with other case-control studies conducted in other developed countries [16, 23, 31]. However, multivariable analyses found that when compared with Pertussis cases, giardiasis cases were 20 times more likely to have been travelling overseas. The most popular countries visited were in South & South-East Asia, West Central Asia/North Africa and Oceania. Overseas travel to endemic regions is widely believed to be the principal risk factor for giardiasis in developed countries. However, due to detection bias associated with physicians testing for giardiasis more commonly among returning travellers, overseas acquired infection rate is likely to be overestimated; and consequently underestimating locally acquired giardiasis [32].

There are some limitations to this study. Although care was taken to recruit controls representative of the source population of cases, some selection bias may exist among controls. There was a larger response rate among older females residents in urban areas in SWS, indicating that women were more likely to respond to the NBH control questionnaire. There was also an underrepresentation of children seen in the NBH controls when compared with Pertussis cases. This selection bias emphasised the sex and age differences between cases and NBH controls and could explain why some exposures were also present among the control group, thus diluting the exposure rates amongst cases. A matched analysis was done to reduce selection bias and improve internal validity, by controlling for the sex, age and region of residence differences between cases and NBH controls. The matched design reduced the risk of error from the confounding effect of age, sex and location but due to the resulting close matching on these variables, their effects on giardiasis risk could not be assessed. However, controlling for these well-known confounders was valuable as it allowed the assessment of other risk factors without their confounding influences. Admission risk bias is a potential problem with Pertussis cases, which were selected based on being a group of patients available through NCIMS, did not have gastrointestinal symptoms or diagnosed with giardiasis and hence they may have a different exposure profile to the general population. Since giardiasis cases matched to pertussis cases were quite similar in sex distribution, there was no association and hence no further need for controlling this variable. Like most studies that utilises surveillance data as a sampling frame, only symptomatic *G. duodenalis* cases that sought medical attention and had a positive laboratory test were included in the study. This means that this study represents only a proportion of the overall burden of the disease in the community. Cases with undiagnosed and asymptomatic giardiasis would not have been considered. Therefore, this study cannot be generalised to all of Australia and must be interpreted in the context of these limitations.

## Conclusion

The study showed an increased risk of giardiasis in children aged under 5 years, amongst individuals who have a household member diagnosed with a gastrointestinal illness and have contact with domestic animals, wildlife or livestock. The study also found that cases who travelled overseas were at a greater risk of infection. There is a need to educate residents living in urban areas in SWS on the potential of person-to-person transmission of giardiasis; particularly if a household member is ill with gastroenteritis. Targeted intervention and health messages are needed for the parents/carers of younger children especially during high-risk seasons such as warmer months, with emphasis on potential risks and appropriate hygiene practices when visiting farms and wildlife parks or where contact with animals is to be expected. Likewise, people travelling overseas to endemic countries should be appropriately informed of the risks and possible control strategies that can be implemented. This study illustrates the value of continuing to monitor giardiasis in south-western Sydney and other parts of NSW and recommends further studies to examine the genotypes in circulation and their potential for zoonotic transmission.

## Supplementary material

For supplementary material accompanying this paper visit https://doi.org/10.1017/S0950268818002637. click here to view supplementary material
