# Supplementary material for: Epidemiology and associated risk factors of giardiasis in a peri-urban setting in New South Wales Australia
Source: Epidemiol Infect. 2018 Sep 28;147:e15. doi: 10.1017/S0950268818002637 (PMC6520257; doi:10.1017/S0950268818002637)
Supplement: Supplementary file 1 [file S0950268818002637sup001.zip › S0950268818002637sup001/S0950268818002637sup001.docx]

Epidemiology and Infection

Epidemiology and associated risk factors of giardiasis in a peri-urban setting in New South Wales Australia

P. ZAJACZKOWSKI, S. MAZUMDAR, S. CONATY, J. T. ELLIS, S. M. FLETCHER-LARTEY

Supplementary Material:

Supplementary Figure S1. A map of the South-Western Sydney Local Health District (SWSLHD) region.


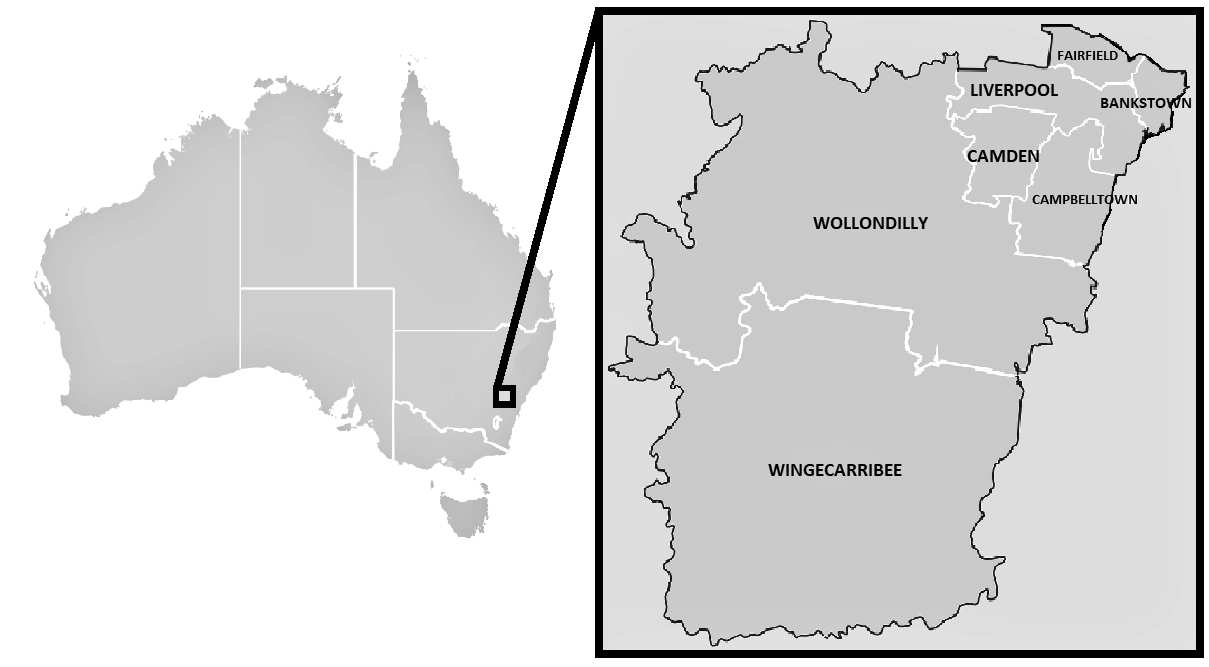


The south-western Sydney (SWS) region includes the following Local Government Areas (LGA); Fairfield, Bankstown, Liverpool, Camden, Campbelltown, Wollondilly and Wingecarribee.

Image adapted from: NSW Government Health. *South Western Sydney* (http://www.health.nsw.gov.au/lhd/pages/swslhd.aspx.). Accessed 20 February 2017.

Supplementary Figure S2. Google map tools used for neighbourhood (NBH) controls.


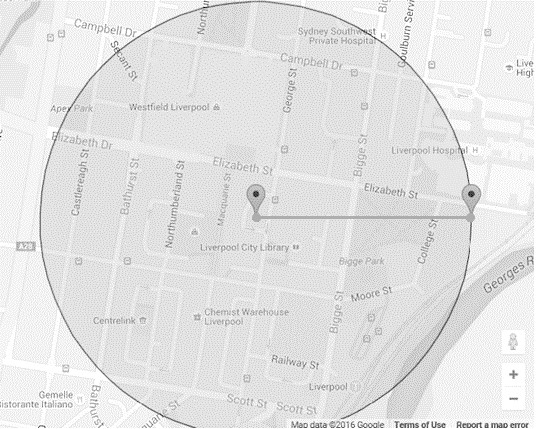


An example of the Google map tools used when drawing a 500m or 5km radius around a cases’ address. The centre point here is the SWSLHD Public Health Unit (PHU).
