# Supplementary material for: Epidemiology and associated risk factors of giardiasis in a peri-urban setting in New South Wales Australia
Source: Epidemiol Infect. 2018 Sep 28;147:e15. doi: 10.1017/S0950268818002637 (PMC6520257; doi:10.1017/S0950268818002637)
Supplement: Supplementary file 5 [file S0950268818002637sup001.zip › S0950268818002637sup001/Giardiasis_MASTER _questionnaire_CASE.odt]

Epidemiology and Infection

Epidemiology and associated risk factors of giardiasis in a peri-urban setting in New South Wales Australia

P. ZAJACZKOWSKI, S. MAZUMDAR, S. CONATY, J. T. ELLIS, S. M. FLETCHER-LARTEY

Supplementary Material: Giardiasis Case-Control Study – Case Questionnaire

Intro: I am >Your Name <, calling from the Public Health Unit, Liverpool. The SWSLHD Public Health Unit is responsible for the investigation and follow-up of cases of giardiasis. Giardiasis is notified to the Public Health Unit by laboratories under the Public Health Act. The Public Health Unit is currently conducting a special project looking into risk factors for developing giardiasis and we have included some questions about possible sources through which people can be exposed. We would like to invite you to participate in a survey. The information you provide will help us to better understand how people become infected with Giardia and identify areas where people may be more at risk for infection so we can make develop strategies to prevent its spread. The information you provide will be treated confidentially and any reports we produce will not include your name or identify you in any way. If you decide not to answer the questions you will not have to provide a reason as to why you don’t. This will not affect you or any present or future treatment/ services provided to you by the Public Health Unit or the health services. You are also free to withdraw your consent and discontinue the interview at any stage. The questionnaire will take approximately 15 minutes to complete. If you’re happy to answer the questions, I would like to proceed with the survey questions. 

☐ Verbal Consent 	    	☐ Parental Consent provided for child <17yrs   	      	☐ Declined  

GIARDIASIS CASE CONTROL STUDY- CASE QUESTIONNAIRE

SECTION A. CLINICAL INFORMATION

The questions in this section relate to your diagnosis and experience with giardiasis.

-  Approximately when were you/ your child diagnosed with giardiasis (remind of date if can’t remember)

- What symptoms did you/ your child have? (Select all that apply)

| Symptoms  (Tick all that apply) | Select | Symptoms continued  (Tick all that apply) | Select |
| --- | --- | --- | --- |
| No or Non-specific Symptoms (Go to Q. 4) |  | Bloating |  |
| Foul-smelling stools |  | Constipation |  |
| Dehydration |  | Diarrhoea or loose stools |  |
| Flatulence or passing wind |  | Myalgia or Muscle pain |  |
| Weakness, excessive tiredness |  | headache |  |
| Nausea |  | Weight loss |  |
| Concentration problems |  | Abdominal cramps |  |
| Other symptoms (specify) |  |  |

- What date did your/ your child’s first symptom(s) appear?  

| Did you/ your child receive any treatment for your symptoms/ diagnosis? | Yes | No |
| --- | --- | --- |
| No, not treated |  |  |
| Treated by GP |  |  |
| Treated in hospital and sent home same day |  |  |
| Treated in hospital and admitted |  |  |
| Over the counter medicine/ home remedies / alternative treatment |  |  |

- During the three months prior to your illness, have you/ your child had any illness or did you/ your child undergo any treatment that has compromised your immunity? (E.g. Cancers, Chemotherapy, HIV etc).

Yes    ☐ 				No   ☐  				Don’t know/ Refuse to answer   ☐

SECTION B. EXPOSURE INFORMATION

The questions in this section relate to activities you/ your child may have been involved in during the three month period before this illness/ diagnosis. 

| During the three month before you/ your child were diagnosed with giardiasis, did you engaged in the following (high risk) occupation(s)? | Yes | No |
| --- | --- | --- |
| Aged Care Worker |  |  |
| Child Care/ Family Dare Care Worker (for child: attended child care) |  |  |
| Food Handler (Chef, Cook, Waiter, Restaurant Cleaner) |  |  |
| Health Care Worker |  |  |
| Nursing Mother (or nurses child that’s not potty trained) |  |  |
| Farm animal handler/ Veterinarian |  |  |
| Other High Risk Occupation (Specify) |  |
| No High Risk Occupation/ Don’t Know |  |  |

6b. If any high risk occupation selected at Q 6, indicate the last date when you/ your child was there. 

Date ___/____/____

- During the period of interest, did you/ your child travel outside of NSW and within Australia?

Yes    ☐ 				No   ☐  			Don’t know/ Refuse to answer   ☐

7b. If yes, indicate places visited (State/Territory).  __________________________________________________________

- During the period of interest, did you/ your child travel overseas and outside Australia?

        Yes    ☐ 				No   ☐  			Don’t know/ Refuse to answer   ☐	

8b. If yes, please state country visited.

__________________________________________________________

8c. Did you visit your country of birth or your parent’s country of birth?

__________________________________________________________

- During the period of interest, did you/ your child go on a camping or caravanning trip?

Yes    ☐ 				No   ☐  			Don’t know/ Refuse to answer   ☐	

9b. If yes, where to?

__________________________________________________________

-  Indicate if any of the following related to you/ your child in the three months before the diagnosis. . If you wish not to answer any question, please select NR for ‘No response’

| Household Information | Yes | No | NR |
| --- | --- | --- | --- |
| Member(s) of household diagnosed with gastrointestinal illness? (If yes, how many?) |  |  |  |
| Member of household  diagnosed with giardiasis |  |  |  |
| Household members attend Child Care regularly |  |  |  |
| Water Information |  |  |  |
| Drank unfiltered or non-boiled tap water |  |  |  |
| Drank filtered tap water |  |  |  |
| Swimming in any type of swimming pool (chlorinated, salt-water and fresh-non-chlorinated water) |  |  |  |
| Swimming in rivers, lakes or lagoons, ponds |  |  |  |
| Swimming in the ocean |  |  |  |
| Sydney Water connect to your home |  |  |  |
| Roof-harvested rain water to your home |  |  |  |
| Bore water or shallow well used in your home |  |  |  |
| Tank water used in your home |  |  |  |
| Storage of water in jars, bottles, cisterns at home. |  |  |  |
| Sanitation and Hygiene |  |  |  |
| Always wash hands before eating? |  |  |  |
| Always wash hands after playing with animals? |  |  |  |
| Biting of finger nails sometimes? |  |  |  |
| Changed nappies of child/children sometimes? |  |  |  |
| Walk bare footed outdoors sometimes? |  |  |  |
| Engaged in any sexual activity with contact with faeces? |  |  |  |
| Onsite septic system at home |  |  |  |
| Outdoor latrine on premises |  |  |  |
| Applied recycled waste water at home/ work |  |  |  |
| Applied sewage/waste water or bio-solids to kitchen garden/ farm |  |  |  |
| Animal Related |  |  |  |
| Own domestic pets (dogs, cats, lizards, pigs, mice etc (indicate which) |  |  |  |
| Contact with farm / domestic animals (If  yes, indicate which) |  |  |  |
| Visited farm, zoo, wildlife park (if yes, where?) |  |  |  |
| Wildlife in close proximity to house (kangaroos, possums, etc) |  |  |  |
| Food Related |  |  |  |
| Consumed unwashed fruits |  |  |  |
| Consumed unwashed vegetables |  |  |  |
| Grow own veggies and fruits |  |  |  |
| Consumed fish caught from local river / pond?. |  |  |  |
| Daily consumer of green salad/lettuce |  |  |  |
| Daily consumer of sushi, other raw seafood dishes (E.g. Sashimi, Kokoda, Oka and ceviche) |  |  |  |
| Other exposure (specify) |  |  |

SECTION C. BACKGROUND INFORMATION

The following questions are about the person who was diagnosed with giardiasis. 

- Case sex: 	

Male    ☐ 			Female    ☐

- Residential Address (Unit / Street Address, City/ Suburb, Postcode) 

- What was the person’s age at their last birthday? 

- What is the language mainly spoken at home? 

- In which Country were you/ your child born?  

- What is you/ your child’s occupation /profession/ main type of job you do? 

- 

| Do you/ your child identify as being of Aboriginal or Torres Strait Islander origin? | Select One |
| --- | --- |
| Not Aboriginal And Torres Strait Islander |  |
| Aboriginal but not Torres Strait Islander |  |
| Torres Strait Islander but not Aboriginal |  |
| Both Aboriginal And Torres Strait Islander |  |
| Not Stated |  |

| How many persons in the following age groups live in your household?   (Indicate the number for all that apply) | No of people in each selected age group |
| --- | --- |
| Under 5 years old |  |
| 5-9 years |  |
| 10-14 years |  |
| 15-19 years |  |
| 20-44 years |  |
| 45-64 years |  |
| 65 and older |  |

| What is the highest level of education you have achieved? (If the case under 12 years old, this info should be provided for the person answering the survey/ head of household). |  |
| --- | --- |
| Had no formal education |  |
| Primary or elementary school (Year K-6 or equivalent) |  |
| Secondary school (Year 7-12 or equivalent) |  |
| Vocational (e.g. TAFE or skills training) |  |
| University |  |
| Other form of education (specify) |  |

END OF SURVEY

This report requires two controls (that is, people who haven’t had giardiasis in the past three months). If possible, can you recommend two friends who live in your local council area (such as Bankstown, Camden, Campbelltown, Fairfield, Liverpool, Wingecaribbe, Wollondilly) but are not residents on your street? You can provide their number or pass on my number to them; 02 8778 0855.

The South Western Sydney Local Health District Public Health Unit would like to thank you for taking the time to complete this survey. We may need to contact you if we have any further questions. If you are happy to be contacted with any further questions, please provide your most appropriate contact details below.

Name: 		___________________________________

Contact email:	____________________________ Contact phone: ________________________

Are you happy to be contacted if we are doing other surveys in the future? (If yes, record this information in the study data tracking spreadsheet) and NOT on this form.
