# Supplementary material for: Epidemiology and associated risk factors of giardiasis in a peri-urban setting in New South Wales Australia
Source: Epidemiol Infect. 2018 Sep 28;147:e15. doi: 10.1017/S0950268818002637 (PMC6520257; doi:10.1017/S0950268818002637)
Supplement: Supplementary file 5 [file S0950268818002637sup001.zip › S0950268818002637sup001/Giardiasis_MASTER _questionnaire_CONTROL.odt]

Epidemiology and Infection

Epidemiology and associated risk factors of giardiasis in a peri-urban setting in New South Wales Australia

P. ZAJACZKOWSKI, S. MAZUMDAR, S. CONATY, J. T. ELLIS, S. M. FLETCHER-LARTEY

Supplementary Material: Giardiasis Case-Control Study – Control Questionnaire 

The SWSLHD Public Health Unit (PHU) is responsible for the investigation and follow-up of cases of giardiasis. Giardiasis is notified to the Public Health Unit by laboratories under the Public Health Act. The Public Health Unit is currently conducting a special project looking into risk factors for developing giardiasis and we have included some questions about possible sources through which people have been exposed. We would like to invite you to participate in the survey. This survey is anonymous and participation is voluntary. You will not be identified in any information that we publish or release. If you decide not to answer the questions you will not have to provide a reason as to why you don’t. 

We would like to collect information about the person in your household who will have the next birthday. By completing this questionnaire you are indicating your consent to participate in this research. If you prefer someone from the Public Health Unit to ask you the questions, please call 02 8778 0855 and ask to speak with someone about the Giardia study. Quote the Control ID number at the top of this page. You may choose to email [swsphu.id@sswahs.nsw.gov.au](mailto:swsphu.id@sswahs.nsw.gov.au) with your telephone number and someone will call you. 

| Who is the person in your household with the next birthday? Write their First Name below | Tick the box beside the description that represents the person with the next birthday in your household. Parent must Consent if under 17 years of age. |
| --- | --- |
|  | ☐  Is this person 12 years or younger? Parent to complete the survey on the child’s behalf. | ☐  Is this person 13 to 16 years of age? With parent’s permission, this child can complete the questions on their own. | ☐  Is this person 17 years or older?  This person should complete the questions on their own. |

I am completing this form on behalf of my child who is 12 years or younger (Please ignore if not applicable)   ☐

GIARDIASIS CASE CONTROL STUDY- CONTROL QUESTIONNAIRE

SECTION A. CLINICAL INFORMATION

The first questions are about your/your child’s current health status. 

- In the past three months, have you/ your child been diagnosed with giardiasis (Intestinal parasites that cause gastro)?

No   ☐  			Yes ☐ (If yes, double check if name is on NCIMS list).

- In the last three weeks have you had any of the following symptoms? (Select all that apply)

| Symptoms  (Tick all that apply) | Select | Symptoms continued  (Tick all that apply) | Select |
| --- | --- | --- | --- |
| No or Non-specific Symptoms (Go to Q. 4) |  | Bloating |  |
| Diarrhoea or loose stools |  | Constipation |  |
| Dehydration |  | Foul-smelling stools |  |
| Flatulence (passing wind) |  | Myalgia or Muscle pain |  |
| Weakness, excessive tiredness |  | headache |  |
| Nausea |  | Weight loss |  |
| Concentration problems |  | Abdominal cramps |  |
| Other symptoms (specify) |  |  |

- If yes, approximately when (what date) did your/ your child’s first symptom(s) appear?  (If there were no symptoms, put N/A).

| In the past three months, did you/ your child receive treatment for giardiasis symptoms/ diagnosis? | Select |
| --- | --- |
| No, not treated |  |
| Yes, Treated by GP (if yes, END interview here) |  |
| Yes, Treated in hospital and sent home same day (if yes, END interview here) |  |
| Yes, Over the counter medicine/ home remedies / alternative treatment (if yes, END interview here) |  |

IF you/ your child has been diagnosed with giardiasis in the past three months, please STOP here & send your responses to the Public Health Unit.

- During the past three months, have you/ your child had any illness or have you/ your child undergone any treatment that has lowered your immunity? (For example Cancers, Chemotherapy, HIV etc).

Yes    ☐ 				No   ☐  			Don’t know/ prefer not to say    ☐

SECTION B. EXPOSURE INFORMATION

The questions in this section relate to activities you may have been involved in during the past three month period. 

| During the past three months, did you/ your child engaged in the following (high risk) occupation(s)? (For children attending child care, select option2 ) | Select one |
| --- | --- |
| Aged Care Worker |  |
| Child Care/ Family Day Care Worker (or Child attending child care) |  |
| Food Handler (Chef, Cook, Waiter, Restaurant Cleaner) |  |
| Health Care Worker |  |
| Nursing Mother (or nurses child that’s not potty trained) |  |
| Farm animal handler/ Veterinarian |  |
| Other High Risk Occupation (Specify) |  |
| No High Risk Occupation/ Don’t Know |  |

6b. If you have ticked any of the occupations at question 6 above, when was the last date when you/ your child was there. 	Date ___/____/____

- During the past three months, did you/ your child travel outside of NSW and within Australia?

Yes    ☐ 				No   ☐  		Don’t know/ prefer not to say    ☐

7b. If yes, Please state all the places where you have travelled to (State/Territory). 

__________________________________________________________

- During the period of interest, did you/ your child travel overseas and outside Australia?

        Yes    ☐ 				No   ☐  			Don’t know/ Refuse to answer   ☐	

8b. If yes, please state country visited.

__________________________________________________________

8c. Did you visit your country of birth or your parent’s country of birth?

__________________________________________________________

- During the period of interest, did you/ your child go on a camping or caravanning trip?

Yes    ☐ 				No   ☐  			Don’t know/ Refuse to answer   ☐	

9b. If yes, where to?

__________________________________________________________

-  Please tick Yes or No if any of the following applied to you/ your child in the previous three months (Select all that apply). If you wish not to answer any question, please select NR for ‘No response’ (Select all that apply).

| Household Information | Yes | No | NR |
| --- | --- | --- | --- |
| Member(s) of household were diagnosed with gastrointestinal illness? (If yes, how many?) _________________________ |  |  |  |
| Member of household  were diagnosed with giardiasis? |  |  |  |
| Household members attend Child Care regularly? |  |  |  |
| Water Information |  |  |  |
| Drink unfiltered or non-boiled tap water? |  |  |  |
| Drank filtered tap water? |  |  |  |
| Swimming in any type of swimming pool (chlorinated, salt-water and fresh-non-chlorinated water)? |  |  |  |
| Swimming in rivers, lakes or lagoons, ponds? |  |  |  |
| Swimming in the ocean? |  |  |  |
| Sydney Water connected to your home? |  |  |  |
| Roof-harvested rain water to your home? |  |  |  |
| Bore water or shallow well water used in your home? |  |  |  |
| Tank water used in your home? |  |  |  |
| Temporary storage of drinking water in jars, bottles, cisterns at home? |  |  |  |
| Sanitation and Hygiene |  |  |  |
| Always wash hands before eating? |  |  |  |
| Always wash hands after playing with animals? |  |  |  |
| Biting of finger nails sometimes? |  |  |  |
| Changed nappies of child/children sometimes? |  |  |  |
| Walk bare footed outdoors sometimes? |  |  |  |
| Engaged in any sexual activity with contact with faeces? |  |  |  |
| Onsite septic system at home? |  |  |  |
| Outdoor latrine on premises? |  |  |  |
| Recycled waste water used at home/ work? |  |  |  |
| Applied waste water or sewage compost to kitchen garden/ farm |  |  |  |
| Animal Related |  |  |  |
| Own domestic pets (dogs, cats, lizards, pigs, mice? (indicate which) |  |  |  |
| Contact with farm / domestic animals/ wildlife? (indicate which) |  |  |  |
| Visited farm, zoo, Wildlife Park? (indicate where) |  |  |  |
| Wildlife in close proximity to house (kangaroos, possums, etc)? |  |  |  |
| Food Related |  |  |  |
| Consumed unwashed fruits? |  |  |  |
| Consumed unwashed vegetables? |  |  |  |
| Grow own veggies and fruits? |  |  |  |
| Consumed fish caught from Local River / pond? |  |  |  |
| Daily consumer of green salad/lettuce? |  |  |  |
| Daily consumer of sushi, other raw seafood dishes (E.g. Sashimi, Kokoda, Oka and ceviche)? |  |  |  |
| Other exposure (please specify)? |  |

SECTION C. BACKGROUND INFORMATION

Please complete the following questions about yourself/your child.

- Your/ your child’s Sex: 	

Male    ☐ 			Female    ☐

- Residential Address (Unit / Street Address, City/ Suburb, Postcode) 

- How old were you/ your child at your last birthday? 

- What is the language mainly spoken at home? 

- In which Country were you/ your child born?  

- What is you/ your child’s occupation /profession/ main type of job? 

- 

| Do you/ your child identify as being of Aboriginal or Torres Strait Islander origin? | Select One |
| --- | --- |
| Not Aboriginal And Torres Strait Islander |  |
| Aboriginal but not Torres Strait Islander |  |
| Torres Strait Islander but not Aboriginal |  |
| Both Aboriginal And Torres Strait Islander |  |
| Not Stated |  |

| How many persons in the following age groups live in your household?   (Indicate the number for all that apply) | No of people in each selected age group |
| --- | --- |
| Under 5 years old |  |
| 5-9 years |  |
| 10-14 years |  |
| 15-19 years |  |
| 20-44 years |  |
| 45-64 years |  |
| 65 and older |  |

 

| What is the highest level of education you/ your child have achieved? (If the case is under 12 years old, this info should be provided for the person answering the survey/ head of household). |  |
| --- | --- |
| Had no formal education |  |
| Primary or elementary school (Year K-6 or equivalent) |  |
| Secondary school (Year 7-12 or equivalent) |  |
| Vocational (e.g. TAFE or skills training) |  |
| University |  |
| Other form of education (specify) |  |

END OF SURVEY

The South Western Sydney Local Health District Public Health Unit would like to thank you for taking the time to complete this survey. The information you have provided will help us to better understand how and where people are exposed to giardiasis infections and how we can control its spread in the community. We may need to contact you if we need to clarify any of this information.

If you are happy to be contacted with any further questions, please provide your most appropriate contact details below.

Name: 		___________________________________

Contact email:	____________________________ Contact phone: ________________________

PLEASE RETURN COMPLETED SURVEY BY POST TO: Public Health Unit, P.O. Box 38, Liverpool, NSW 1871.

Are you happy to be contacted if we are doing other surveys in the future? (If yes, record this person’s contact information in the study data tracking spreadsheet) and NOT on this form.
